# Neonatal imitation predicts infant rhesus macaque (*Macaca mulatta*) social and anxiety-related behaviours at one year

**DOI:** 10.1038/srep34997

**Published:** 2016-10-11

**Authors:** Stefano S. K. Kaburu, Annika Paukner, Elizabeth A. Simpson, Stephen J. Suomi, Pier F. Ferrari

**Affiliations:** 1 Department of Population Health and Reproduction, University of California, Davis, California, USA; 2 Eunice Kennedy Shriver National Institute of Child Health and Human Development, National Institutes of Health, Poolesville, Maryland, USA; 3 Department of Psychology, University of Miami, Coral Gables, Florida, USA; 4 Dipartimento di Neuroscienze, Università di Parma, Parma, Italy; 5 Institut des Sciences Cognitives Marc Jeannerod CNRS/Université Claude Bernard Lyon 67 Bd Pinel, 69675, Bron, Cedex, France

## Abstract

The identification of early markers that predict the development of specific social trajectories is critical to understand the developmental and neurobiological underpinnings of healthy social development. We investigated, in infant rhesus macaques (*Macaca mulatta*), whether newborns’ capacity to imitate facial gestures is a valid predictive marker for the emergence of social competencies later in development, at one year of age. Here we first assessed whether infant macaques (N = 126) imitate lipsmacking gestures (a macaque affiliative expression) performed by a human experimenter in their first week of life. We then collected data on infants’ social interactions (aggression, grooming, and play) and self-scratching (a proxy indicator of anxiety) at 11–14 months when infants were transferred into a new enclosure with a large social group. Our results show that neonatal imitators exhibit more dominant behaviours, are less anxious, and, for males only, spend more time in play at one year old. These findings suggest that neonatal imitation may be an early predictor of infant sociality and may help identify infants at risk of neurodevelopmental social deficits.

In humans, there are large inter-individual differences in the ability to establish and maintain social relationships, which have profound consequences on psychological well-being. Individuals with good social skills experience higher levels of happiness, self-esteem, and quality of life^1^. In contrast, those who lack these skills tend to develop a variety of problems, including social anxiety and phobia, depression, and loneliness^2^. The identification of early behavioural markers that can anticipate specific developmental trajectories has, therefore, become increasingly important to determine appropriate early intervention strategies for socially impaired individuals^3^.

Infants’ ability to imitate facial gestures in their first weeks of life is thought to be one of the earliest measures of the presence of inter-individual differences in social skills^4^. Newborns as young as 42 min are able to imitate open mouth and tongue protrusion gestures^5^, as well as facial expressions like happiness, sadness, and surprise^6^. Neonatal imitation has been suggested to enrich infants’ understanding of people^7^, and strengthen their bonds with caregivers^8^. Although the presence of this phenomenon at the population level is still debated^9^, there is clear evidence of ample inter-individual variability in neonatal imitative responses^6^,^10^. Heimann and colleagues^10^ for instance, showed that 44% of infants at 2–3 days of age imitate mouth opening and 61% imitate tongue protrusion, while Field^6^ found that approximately one-third of human neonates (average age 36 hours) exhibited strong imitation skills, while one-third showed weak imitative responses, and one-third did not imitate the model.

Neonatal imitation is a phenomenon that occurs also in some non-human primates (NHP), namely chimpanzees (*Pan troglodytes*^11^), and rhesus macaques (*Macaca mulatta*^12^,^13^). As in human infants, even macaque infants show large inter-individual differences in their imitative abilities^14^. Although neonatal imitation has been suggested to be an early foundation for infants’ subsequent cognitive and social development^15^, there are few empirical studies that have investigated this idea. In humans, for instance, compared to non-imitators, infants who are imitators at 2–3 days and 3 weeks old show lower rates of gaze aversion at 3 months old when interacting with their mothers^4^. Similarly, in rhesus macaques, neonatal imitators look more at faces, especially at the eye region, of conspecifics at 10–28 days old^16^ and are better at gaze following at 7 months old^13^ than non-imitators, suggesting that imitators may more readily read social cues^16^. In the first week of life, imitators, compared to non-imitators, appear to (1) be more attentive during neonatal imitation assessments^17^, (2) better remember social partners^18^, and (3) exhibit superior delayed imitation^19^, suggesting imitators may be more socially advanced than non-imitators. It remains largely unclear, however, whether neonatal imitation is simply the expression of a general behavioural response that is contingent with the context and the stage of development, or whether it represents a basic biological predisposition that could be considered as a trait with clear connections with other traits. In the latter case, it is expected that neonatal imitation is not only linked to other behaviours that are associated with social perception and social interest, but it is expected that it may predict social development at later ages. However, this critical information is missing from the current literature. To fill this gap, we took advantage of the described interindividual variability in neonatal imitative responses and examined whether infant rhesus macaques who imitate facial gestures in their first week of life displayed greater social skills than non-imitators at one year old. We also examined whether imitators exhibit lower anxiety levels in a novel social context since, in both humans and NHP, individuals with poor social skills tend to exhibit increased anxiety and stress^20^,^21^.

Rhesus monkeys are an ideal study model in which to investigate this issue. Similar to humans, mother and infant macaques form strong bonds, with mothers engaging in a variety of communicative gestures with infants, such as lipsmacking, exaggerated head movements, and mutual gazing^22^,^23^. These exchanges seem to resemble the ‘motherese’ that human mothers display towards their infants^22^. As in humans, the quality of this early social enviroment can have profound consequences on infant’s cognitive and social development, resulting in large variation in social skills in adolescence and adulthood^24^,^25^.

Here we predict that at approximately 1 year old, neonatal imitators, compared to non-imitators, will:

1. spend more time in social play, as play behaviour functions as a way for infants to learn species-specific signals^26^, form alliances and assess and manipulate social relationships^27^;
2. spend more time in social grooming, which, in NHP, is a means by which individuals strengthen social relationships^28^;
3. display a greater frequency of dominant behaviours measured as aggression directed to peers as dominant behaviour is commonly linked to an individual’s ability to attain higher ranks^29^; and
4. show lower rates of self-scratching, which, in NHP, is considered a proxy indicator of anxiety^30^.

## Results

We assessed neonatal imitation in the first week of life on 126 newborn rhesus macaques, and infants were then classified as either lipsmacking (LPS) imitators or non-imitators (see Methods). Of the 126 infants tested, 48% were classified as LPS imitators. We then measured macaque infants’ social behaviours at approximately one year of age, when placed in a large group with same-aged peers. We ran Linear Mixed Model (LMM) analysis to assess whether infants’ imitation category in the first week of life predicted the time infants spent in play and social grooming as well as infants’ rates of aggression directed at peers and self-scratching at 1 year of age.

### Neonatal imitation and social behaviours at 1 year

**Figure 1:**
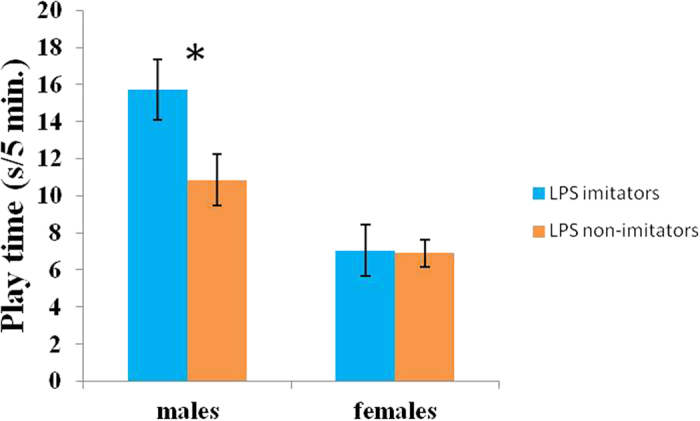
Effect of lipsmacking (LPS) neonatal imitation category on play time at one year old for each sex. Male imitators spent more time playing with their peers than non-imitators. There was no difference in play time between female imitators and non-imitators. *p < 0.05.

The amount of time infants spent playing with peers at 1 year of age was significantly predicted by both sex (Estimate ± SE: −0.952 ± 0.204, t = −4.665, p < 0.001, LRT = 20.42, Table 1) and the interaction between sex and neonatal LPS imitation category (Estimate ± SE: −0.777 ± 0.263, t = −2.957, p = 0.004, LRT = 8.41, Table 1): male infants played significantly more (mean ± SD: 13.22 s ± 9.34 s) than females (6.97 s ± 5.60 s). Follow up t-test showed that male imitators played more than male non-imitators (t(80) = 2.265, p = 0.027, d = 0.53, Fig. 1), which is consistent with *prediction 1*. Contrary to *prediction 2*, however, no effect of LPS imitation category was found on the amount of grooming exchanged (imitators: 5.05 s ± 6.29 s; non-imitators: 5.66 s ± 5.57 s, Table 1).

**Figure 2:**
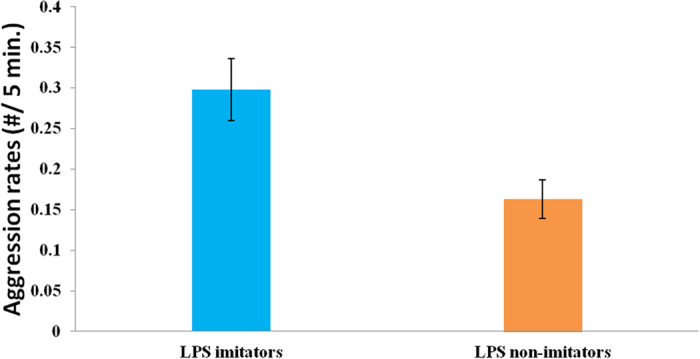
Effect of lipsmacking (LPS) neonatal imitation category on aggression rates. LPS imitators directed more aggression to peers compared to non-imitators.

We found support for *prediction 3*: our LMM model revealed that LPS imitation significantly predicted rates of aggression directed at peers (Estimate ± SE: 0.089 ± 0.043, t = 2.069, p = 0.039, LRT = 4.31, Table 1). LPS imitators displayed significantly higher rates of aggression compared to non-imitators (Fig. 2).

### Neonatal imitation and rates of self-scratching at 1 year

**Figure 3:**
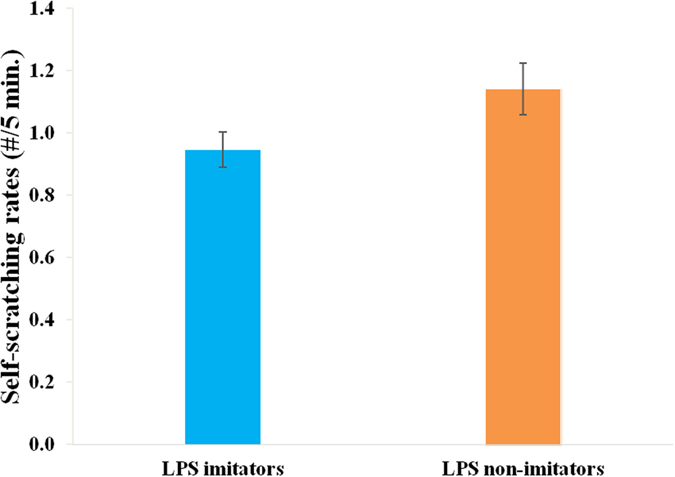
Effect of lipsmacking (LPS) neonatal imitation category on self-scratching rates. Imitators self-scratched less frequently than non-imitators.

We found that LPS imitation significantly predicted rates of self-scratching (Estimate ± SE: 0.087 ± 0.044, t = −1.981, p = 0.048, LRT = 3.93, Table 2), with LPS imitators showing significantly lower rates of self-scratching than LPS non-imitators (Fig. 3), suggesting that imitators exhibited lower anxiety levels than non-imitators.

## Discussion

Our results build up on previous reports that infant rhesus monkeys display large inter-individual variability in their neonatal imitative abilities, with nearly half of the infants showing the capacity to imitate LPS gestures^13^,^14^,^16^,^17^,^18^,^19^. However, by using a large cohort of infants, our work provides the first evidence that infants’ ability to match a caregiver’s gestures predicts infant social behaviour and temperament at one year old, as LPS imitators exhibited greater dominance behaviour (expressed through increased aggression rates) and lower anxiety levels, and male imitators tended to play more than non-imitators at 1 year of age. These results indicate that neonatal imitative abilities not only reflect infants’ social skills or interest in the first weeks or months of life, as previous work in human and macaque has shown^10^,^13^,^14^,^16^,^19^, but also reflect a longer developmental trajectory that encompasses the first year of life.

Both aggressive and play behaviours serve important roles for infants’ development of social skills and establishment in a new social group^26^,^31^. Aggression is commonly used in primates as a way to achieve and maintain high ranks^29^, which ultimately increases individuals’ inclusive fitness. Furthermore, third-party agonistic interventions in aggressive interactions play a fundamental role in stabilizing social groups^32^, as well as in establishing and maintaining dominance relationships and cementing alliances, contributing thereby to the formation of social bonds^33^. Finally, aggression has the fundamental function of obtaining and securing resources for both sexes, such as mates for males and feeding sources for females, which can explain why we did not find sex differences in the rates of aggression infants directed to peers. This finding is consistent with previous studies on rhesus macaques showing that while adult males and females display differences in aggression rates^34^, no sex difference in aggression is apparent in the first three years of life^35^. In free-ranging populations of rhesus monkeys, mother’s rank, and the presence of close relatives have a strong influence on infant’s ability to achieve high-ranks, as dominant females and family members are more likely to support infants than low-ranking females or unrelated group members^36^. Our work suggests that, when infants are raised in the absence of mothers or close relatives, and under homogeneous environmental conditions (except for the difference in rearing condition, which, in our study, did not predict any of the examined behaviours), infants’ biological predisposition to exhibit advanced social skills can be fundamental in the acquisition of dominant positions at least at one year of age (but see Bastian *et al*.^37^). Our work therefore indicates that these greater social skills can already be detected in the first week of life.

By including behavioural patterns typical of ‘serious’ functional contexts, such as mating, agonistic, and anti-predatory behaviours^38^, play behaviour offers a fundamental contribution for infant cognitive and social development across a broad range of animal species^26^. Through play, infants learn context-specific signals^39^, develop coordinate and cooperative skills^40^, and help infants assess the strength of their future competitors^41^. Accordingly, among yellow-bellied marmots (*Marmota flaviventris*), the outcome of play interactions predicts adult dominance rank^42^, while in brown bears (*Ursus arctos*) play behaviour increases infant chances to survive to independence^43^. In humans, peer play among children has been shown to have a strong impact on social development, through the acquisition, for instance, of conflict-resolution and cooperative-learning skills^44^: children who experience peer-rejection, for instance, suffer psychosocial and conduct problems^45^, which can also have negative consequences on children’s learning skills, leading to poor school performance^46^.

One of the core aspects of play, especially in the first years of life, is the ability to imitate peers: the performance of same acts is the predominant mode of social interactions among toddlers, and these imitative exchanges promote continued social interactions by communicating a common understanding of ongoing activities^47^ and are fundamental for the development of more advanced play skills at later ages^48^. Accordingly, children between 4 and 6 years of age tend to be attentive to peers’ activity, and this ability to observe peer behaviour has been found to have positive effects on children’s own performance of these activities^48^. The importance of imitation during social play is also supported by work conducted on NHP: in geladas (*Theropithecus gelada*), and Tonkean macaques (*M. tonkeana*), for instance, play bouts in which participants engage in rapid mimicry of facial expressions tend to last longer than bouts where this form of facial imitation is absent^49^,^50^. This capacity to imitate peers’ behaviours likely originates from newborn’s ability to match caregiver’s behaviours. For instance, infant rhesus macaques who engage in more face-to-face interactions with their caregiver in the first month of life display enhanced social development at later ages^25^.

Notably, children with neurodevelopmental disorders, such as autism spectrum disorder (ASD), exhibit impaired play activity with peers^51^ as well as deficit in imitative abilities^52^. This difficulty of children with ASD to imitate a model’s actions has been shown to be specific to non-meaningful (i.e. non goal-directed) gestures and appears to be due to an impaired selection mechanism (i.e. ‘what’ to imitate) which is related to these children’s impaired capacity to attend social stimuli and recognize intentional actions^52^.

Although our results need to be replicated in humans, our work suggests that these imitation skills can already be detected in newborns, and can be used as reliable indicators of specific developmental outcomes, at least until one year of age in macaques. In other words, in humans, classifying newborns based on their neonatal imitative behaviours may potentially lead to the identification of infants who might exhibit impaired social development. This classification, in turn, may facilitate testing of intervention programs aimed at improving the social development in infants and children who lack social skills. To date, a variety of methods have been developed to treat infants with neurodevelopmental disorders (e.g. ASD), either through pharmacological treatments^53^, or by providing a richer social environment^54^. However, these treatments are generally provided at an advanced developmental stage, when the toddler or child has already been diagnosed with ASD. Therefore, it is crucial to be able to identify early markers that can help detect neurodevelopmental disorders at an earlier developmental stage, when the brain is more plastic, and the course of early behavioural and brain development can be altered^3^. Our work, thus, should encourage more longitudinal studies in humans to assess to what extent children who imitate facial gestures when neonates are also more social in the first years of life (the developmental stage of a one-year old rhesus monkey corresponds approximately to the developmental stage of a four-year old human child^55^).

In the present study, we found the association between neonatal imitative response and play behaviour at 1 year old exclusively in males. In rhesus macaques, while females remain in their natal group, males transfer to neighbouring groups at approximately 3 years old. This process of migration is risky, as males face high predation risks and need to successfully integrate and be accepted into a new group. Accordingly, males suffer higher mortality than females, especially when they transfer to other groups^56^. These conditions might have selected males to have evolved a specific set of social skills from the earliest weeks of life than females. While we found no sex differences in neonatal imitation rates, a previous study reported female infant macaques appear more socially interested than males between 2–5 weeks of age, looking more to faces, especially the eyes, and exhibiting more affiliative behaviours towards human caretakers^57^. Together, with the present findings, these data suggest there may be sex differences in both early and later social trajectories. Future work in humans should explore the presence of early sex-specific behavioural markers that can help detect the development of neurodevelopmental disorders, especially for those disorders that are biased towards a specific sex, such as ASD^58^.

Finally, we found that neonatal imitators displayed lower anxiety levels, assessed through the measurement of self-scratching rates, compared to non-imitators. Dettmer *et al*.^59^ showed that relocation to a new enclosure with a larger social group (which includes also mother-reared infants) is a major source of stress for nursery-reared infants within the first year after the relocation. Our results suggest that imitators might be better able to cope and adapt with the new environment and better integrate into the new social group. It is plausible that the mechanism behind imitators’ greater social skills and lower anxiety levels coincide, as a large body of research has consistently found a positive link between peer acceptance and sociality, and a negative association between sociality and anxiety in both human and NHP^21^.

Overall, our study shows that neonatal imitation in rhesus macaques may be employed as an early marker that can help anticipate specific developmental trajectories, as neonatal imitators exhibit more dominant behaviours, are less anxious, and male imitators play for longer than non-imitators. More importantly, our results contribute to a body of research that aims to identify early markers of impaired social and cognitive competencies. While replications in human infants are necessary, we believe this study represents a large step forward, highlighting the utility of considering neonatal imitation as a meaningful, predictive early marker of sociality.

## Materials and Methods

### Subjects and housing conditions

The study was conducted on 126 healthy nursery-reared infants, raised at the Laboratory of Comparative Ethology at the National Institutes of Health in eight cohorts between 2007 and 2014. All procedures described below adhered to the NIH Guide for the Care and Use of Laboratory Animals and were approved by the NICHD Animal Care and Use Committee.

Infants were separated from their mothers on the day of birth, and raised in a nursery following the protocol reported by Shannon *et al*.^60^. In brief, for the first 14 days of life, infants were housed in plastic incubators (51 × 38 × 43 cm), before being moved to cages (65 × 73 × 83 cm) from the third week of life until approximately 8 months old. In both housing conditions, infants were provided with an inanimate cloth-covered surrogate, along with pieces of fabric fleece and various toys. For unrelated studies, infants were randomly assigned to one of two rearing conditions when the youngest infant of the group turned 36 days: some (N = 57) were reared with three to four peers (peer-rearing), while others (N = 69) were reared with their surrogate and were given 2-hr play sessions with three to four peers (surrogate-peer-rearing) each weekday. At about 8 months of age, infants were relocated into a single social group, which included peer-reared, surrogate-peer-reared, and mother-reared infants. The size of this social group ranged between 14 and 59 infants, with an average group size of 40.6 (median: 42) infants. The enclosure consisted of an indoor and outdoor area: the indoor enclosure measured 7.3 × 3.4 × 3.7 m and was equipped with perches, barrels, swings, and wood shavings, while the outdoor area was a circular corn-crib enclosure measuring 5.03 m in diameter by 5.49 m high. All infants had free access to both areas, except when they were partitioned to either enclosure for routine cleaning, or to the inside during inclement weather^59^.

### Neonatal imitation task

Following previous studies^16^,^19^, we tested infants on a neonatal imitation task in their first week of life, two times a day, every other day (days 1–2, 3–4, 5–6, and 7–8), with at least an hour break between each test session. In each test session, infants were held by one experimenter, while a second experimenter presented the stimuli, and a third experimenter was the time-keeper who signaled the start and end time of each phase of the test. We presented infants with two different stimuli, one during each session, at a distance of approximately 30 cm at eye-level with the infant: 1) a lipsmacking gesture (LPS) which is a common affiliative gesture in macaque and consists of the rapid opening and closing of the mouth; and 2) a nonsocial control condition (a white plastic disk with black/red or green/yellow orthogonal stripes slowly rotated clockwise and counter-clockwise). The order in which stimuli were presented remained the same for each infant across days, but was randomized across infants.

The test lasted 3 minutes total: in the first 40 seconds (BASELINE 1) the demonstrator either displayed a calm, neutral facial expression in the LPS condition or held the disk still in the control condition. In the following STIMULUS period, the demonstrator either displayed a lipsmacking gesture, or rotated the disk in the control condition, for 20 seconds. Then, for 20 sec the demonstrator showed again either a neutral facial expression or a still disk. This movement-still face sequence was repeated three times (STIMULUS), with the final still face expression period lasting 40 sec (BASELINE 2). We videotaped all sessions using a Sony Digital Video camcorder (either a ZR600 or HDR-CX560V).

### Social interactions with peers at 1 year

Four observers who were blind to the research questions scored infants’ behaviours in their social group between ~11 months (mean ± SD = 339 ± 41 days) and ~14 months (mean ± SD = 405 ± 58 days) of age, using the J-watcher software. To facilitate data collection, during behavioural observations infants were partitioned in the indoor enclosure. Social interactions and self-directed behaviours were recorded through 5 minutes focal animal sampling, twice a week, once in the morning and once in the afternoon. The following data were collected:

1. *Aggression*: frequency of bites, hair pulls, aggressive chases, threats, hitting, slapping, or displacements.
2. *Play*: duration of play behaviours that included play face, non-aggressive chasing, tagging, swatting, bobbing, biting, pulling, lunging, mouthing, and wrestling (rough and tumble).
3. *Social grooming*: duration of cleaning or manipulating the fur of another individual.
4. *Self-scratching*: frequency count, common usage.

#### Data analysis

For the neonatal imitation assessment, we coded infants’ mouth movements off-line, frame-by-frame (30 frames per second). Coders had at least 6 months of experience with macaque infants, were familiar with their gestures, and were blind to the stimulus presented (disk or lipsmacking gesture). We defined lipsmacking as a high frequency opening and closing of the mouth within 2 seconds. Infants were classified as LPS imitators if they displayed on average across the four testing days higher rates of LPS gestures during the Stimulus period than the Baseline period in the LPS condition, and this increase was larger in the LPS condition than in the disk control condition^16^.

We ran linear mixed model analysis (LMM), using the *lmer* function in the lme4 package implemented in R v3.1.2, in which we included mean duration of play and social grooming and mean rates of aggression given and self-scratching across focal observations as dependent variables in separate models, while LPS imitation category, infant sex, and rearing condition prior to weaning, as well as the interaction between imitation category and sex, were set as fixed factors in all models. All models included the cohort identity as random factor, to control for a potential cohort-effect. All dependent variables were square root transformed to normalize their distribution. We used the *drop1* function to calculate the likelihood ratio test (LRT) to compare the full models (the model with all the fixed effects) to a reduced model (the model without the variable of interest).

## Additional Information

**How to cite this article**: Kaburu, S. S. K. *et al*. Neonatal imitation predicts infant rhesus macaque (*Macaca mulatta*) social and anxiety-related behaviours at one year. *Sci. Rep*. **6**, 34997; doi: 10.1038/srep34997 (2016).

## Figures and Tables

**Table 1:** Results of the LMM model to test the effect of LPS neonatal imitation category, infant sex, rearing condition, and the interaction between LPS neonatal imitation category and sex on social behaviours at one year of age, namely rates of aggression given, play and grooming time.

Independent	Predictor	Estimate	SE	t-value	P
Play	Intercept	3.216	0.250	12.880	
	LPS imitation	0.302	0.201	1.499	0.127
	**Sex**	**−0.952**	**0.204**	**−4.665**	**<0.001**
	rearing	0.272	0.203	−1.340	0.173
	Sex * LPS imitation	**−0.777**	**0.263**	**−2.957**	**0.004**
Grooming	Intercept	1.993	0.285	6.992	
	LPS imitation	−0.217	0.201	−1.079	0.278
	Sex	0.208	0.204	1.020	0.311
	rearing	−0.118	0.203	−0.579	0.552
	Sex * LPS imitation	−0.060	0.251	−0.238	0.806
Aggression given	Intercept	0.351	0.089	3.927	
	**LPS imitation**	**0.089**	**0.043**	**2.069**	**0.039**
	Sex	−0.063	0.044	−1.436	0.150
	rearing	0.044	0.044	1.005	0.307
	Sex * LPS imitation	0.047	0.055	0.853	0.387

Significant predictors (p < 0.05) are shown in bold.

**Table 2:** Results of the LMM model to test the effect of LPS neonatal imitation category, infant sex, rearing condition, and the interaction between LPS neonatal imitation category and sex on rates of self-scratching at one year of age.

Independent	Predictor	Estimate	SE	t-value	P
Self-scratching	Intercept	1.031	0.077	13.497	
	**LPS imitation**	**−0.087**	**0.044**	**−1.981**	**0.048**
	Sex	−0.046	0.044	−1.045	0.292
	rearing	0.016	0.044	0.370	0.706
	Sex * LPS imitation	−0.068	0.055	−1.246	0.212

Significant predictor (p < 0.05) is shown in bold.
